# Assessment of implicit COVID-19 attitudes using affective priming for pro-vaccine and vaccine-hesitant individuals

**DOI:** 10.1177/13591053231176261

**Published:** 2023-06-02

**Authors:** Stefania S Moro, Jennifer K E Steeves

**Affiliations:** York University, Canada

**Keywords:** affective priming, attitude, COVID-19, implicit behaviour, vaccination

## Abstract

The COVID-19 pandemic has resulted in the introduction of pharmaceutical and non-pharmaceutical interventions such as precautionary behaviours. The current study used affective priming to evaluate COVID-19 attitudes in vaccine-hesitant and pro-vaccine participants. Explicitly, both groups rated their overall perception of risk associated with contracting COVID-19 significantly lower compared to their perception of necessary precautions and overall adherence to public health measures. Pro-vaccine participants rated their perception of necessary precautions higher compared to vaccine-hesitant participants. During baseline measures, both groups classified COVID-19 affiliated words as unpleasant. Affective priming was observed for congruent prime-target pleasant and unpleasant word pairs but was not observed for COVID-19 related word pairs. Differences between groups in the perception of necessary public health precautions points to different underlying pathways for reduced perceived risk and lack of affective priming. These results refine previous findings indicating that implicit attitudes towards COVID-19 can be measured using the affective priming paradigm.

The novel coronavirus (COVID-19) outbreak that began in late 2019 has had a devastating impact on all aspects of society worldwide. To date, the pandemic has resulted 616,951,418 confirmed cases and 6,530,281 COVID-19 related deaths across 222 countries, areas, and territories as of October 7, 2022 (World Health Organisation, 2022). Initially, to mitigate this highly contagious and rapidly spreading virus, public health agencies and governments adopted non-pharmaceutical precautionary behavioural interventions such as total or partial lockdowns, social distancing, movement restrictions including closed borders, and face mask mandates as the first line of defence to reduce the spread of COVID-19 (Biscayart et al., 2020; Zaka et al., 2020; Zhao et al., 2020). In addition to non-pharmaceutical interventions, medical technology including effective, safe, and affordable vaccines and later, during the course of the pandemic, antiviral drugs, that were rapidly developed, comprise a promising route to contain the pandemic and at minimum reduce mortality and morbidity rates (El-Elimat et al., 2021). Vaccines have been shown to be a reliable and cost-effective public health tool and are credited with saving millions of lives each year (Ehreth, 2003; El-Elimat et al., 2021; Hajj Hussein et al., 2015; Rodrigues and Plotkin, 2020).

Non-pharmaceutical precautionary behavioural interventions have been scrutinized throughout the COVID-19 pandemic. The implication that precautionary behaviours contribute to increasing the risk for pervasive mental health problems and psychological fear-related responses (for review see: Ehreth, 2003; Rajkumar, 2020; Xiong et al., 2020) has been argued as a negative side-effect that must be balanced throughout each stage of the ongoing pandemic. As a result, behaviours such as ignoring recommendations for social distancing and masking, as well as continuing to travel despite restrictions have been observed. These types of high-risk behaviours contribute towards accelerating the spread of the virus (Amsalem et al., 2021). Conversely, fear-related behaviours, such as extreme avoidance of social contact, contribute to increased risk of mental health problems (Amsalem et al., 2021). Additionally, vaccine hesitancy is a major obstacle hindering vaccine-induced herd-immunity in various populations throughout the world (Coustasse et al., 2021; El-Elimat et al., 2021; MacDonald, 2015; Neumann-Böhme et al., 2020; Schoch-Spana et al., 2021). One of the contributing factors towards vaccine hesitancy is complacency, associated with a low realized risk of the vaccine-preventable disease leading to more negative attitudes towards the vaccines (Al-Mohaithef and Padhi, 2020; El-Elimat et al., 2021; French et al., 2020), as well as concerns about vaccine induced side-effects (see Medeiros et al., 2022 for a review). The combination of high-risk behaviours resulting from low realized risk of the virus and fear-induced behaviours have shaped the short- and long-term course of the pandemic (Amsalem et al., 2021; Cénat et al., 2020; Shultz et al., 2016).

Psychologists have developed methods for evaluating our perception of risk, attitudes, and behaviours towards emotional information content. In everyday life, we evaluate opportunities and risks from our surrounding environment to determine appropriate behaviour (Klauer, 1997). Automatic adaptive responses are generated when environmental stimuli are evaluated as pleasant or unpleasant prior to explicit cognitive analysis of the stimulus (Klauer, 1997). This type of implicit evaluative response has been illustrated by the affective priming paradigm (Draine and Greenwald, 1998; Fazio et al., 1986; Klauer, 1997) that investigates whether the assessment of a first stimulus (the prime) affects the processing of a subsequent stimulus (the target) (for review see: Klauer, 1997; Klauer and Musch, 2003). When a polarized target word (e.g. happiness) is preceded by a congruently-polarized prime word (e.g. peace) rather than an incongruently-polarized prime word (e.g. terror), a facilitation effect is observed in the form of a faster response time (Klauer and Musch, 2003). The affective priming paradigm is an ideal behavioural task that is able to indirectly probe the dynamics of implicit evaluative processing (Klauer and Musch, 2003). This is possible as the affective value of words perceived by a participant reflect the attitude of the participant towards the word, for example positive or negative views of a person, place, thing, or event (Abbassi et al., 2019). The affective priming paradigm has been used in the context of evaluating racial attitudes (Abbassi et al., 2019; Dovidio et al., 1997; Fazio et al., 1995; Wittenbrink et al., 1997), where for example, participants presented with prime words such as *black* or *white* followed by target words that varied in valence and stereotypicality for white people and black people. Black primes resulted in stronger facilitation to negative compared to positive stereotypic attributes, while the opposite was true for white primes (Wittenbrink et al., 1997). Similar studies have been conducted to investigate sexual health behaviours by comparing implicit and explicit attitudes towards casual condom use where attitude priming was not positively correlated with casual condom use (Marsh et al., 2001). Additionally, affective priming has been used to evaluate whether implicit self-esteem reflects explicit self-esteem evaluations based on introspectively inaccessible associations to the self (Spalding and Hardin, 1999; see Greenwald and Farnham, 2000 for a review). It is possible to evaluate attitudes based on the classification of target words into two categories (e.g. pleasant and unpleasant). Prime words, associated with the attitude being investigated are presented prior to the target words which impact the response times to the target word. Prime-target pairs that are affectively congruent reduce response times, whereas prime-target pairs that are incongruent increase reaction times. Observing the resulting reaction time performance allows researchers to infer positive or negative attitudes associated with the prime words. Recently, affective priming was used to evaluate implicit attitudes towards COVID-19 (Moro and Steeves, 2021). Participants were asked to categorize affective target words into pleasant and unpleasant categories. Each trial, participants were presented with congruent and incongruent prime-target pairings, as well as baseline trials. Participants were instructed to respond as quickly and as accurately as possible by ignoring the first word (prime stimulus) and to classify the second word (target) as either pleasant or unpleasant using two corresponding buttons on their keyboard. Explicitly, participants rated their overall risk perception associated with contracting COVID-19 significantly lower compared to their perception of necessary precautions and overall adherence to public health measures (Moro and Steeves, 2021). Despite rating the COVID-19 affiliated words as unpleasant, affective priming was not observed for congruent prime-target COVID-19 affiliated word pairs when compared to congruent prime-target pleasant and unpleasant words (Moro and Steeves, 2021). This study provided quantitative evidence that COVID-19 affiliated words do not invoke the same implicit attitude response as traditional pleasant and unpleasant word stimuli, despite conscious explicit rating of the COVID-19 words as unpleasant.

Non-pharmaceutical behavioural interventions in combination with pharmaceutical interventions have been shown to contribute positively towards reducing the spread of the COVID-19 pandemic. However, the inability or unwillingness by certain members of the general public to participate in the various recommended interventions has shaped the short-and long-term trajectory of the pandemic. The current study, conducted during the fifth COVID-19 wave in Canada, aimed to compare participants who are pro-vaccination and vaccine-hesitant by measuring their implicit and explicit attitudes towards COVID-19. Implicit attitudes were measured through an affective priming task and explicit attitudes were measured through baseline measurements and survey responses, similar to the previous study by Moro and Steeves (2021). We predicted that pro-vaccination participants will explicitly rate their risk perception associated with contracting COVID-19 significantly higher compared to vaccine-hesitant participants. Further, similar to Moro and Steeves (2021) we predicted that all participants would classify COVID-19 associated words as unpleasant during baseline trials. Finally, despite classifying COVID-19 associated words as being unpleasant, we predicted that on one hand, pro-vaccination participants would demonstrate affective priming, therefore indicating that their explicit and implicit attitudes towards COVID-19 are aligned, whereas on the other hand, vaccine-hesitant participants would not show affective priming demonstrating an attitude-behaviour discrepancy. This research examines whether the increased incidence in risky-behaviour is more prevalent in vaccine-hesitant demographic of the population and provides a better understanding of why some individuals across communities might be more willing to engage in precautionary behaviours outlined by their public health agencies to mitigate the spread of COVID-19.

## Methods

### Participants

An a priori power analysis was conducted using G*Power version 3.1 (Faul et al., 2007) for sample size estimation for a repeated-measures, within factors ANOVA. With a significance criterion of α = 0.05 and power = 0.95, the minimum sample size needed for a medium (0.25) effect size is *N* = 44 participants.

#### Vaccine-hesitant participants

Twenty participants with a mean age of 55 years (SD = 6; 15 female; 3 left hand dominant) volunteered to participate in this study in exchange for course credit for an introductory psychology course (if applicable). All participants were self-described as vaccine-hesitant and reported not receiving a primary series of any COVID-19 vaccine (minimum two doses). Those who reported receiving a primary series of a COVID-19 vaccine series reported only doing so based on a government mandate to attend work or school.

#### Pro-vaccine participants

Twenty participants with a mean age of 37 years (SD = 14; 13 female; 4 left hand dominant) volunteered to participate in this study in exchange for course credit for an introductory psychology course (if applicable). All participants were self-described as pro-vaccine and reported receiving at least a primary series of any COVID-19 vaccine.

All participants were self-described as native English speakers living in Canada and were members of the York University community including staff and students. Participants were excluded from this study if they did not report themselves as a native English speaker.

### Stimuli

**Figure 1.**
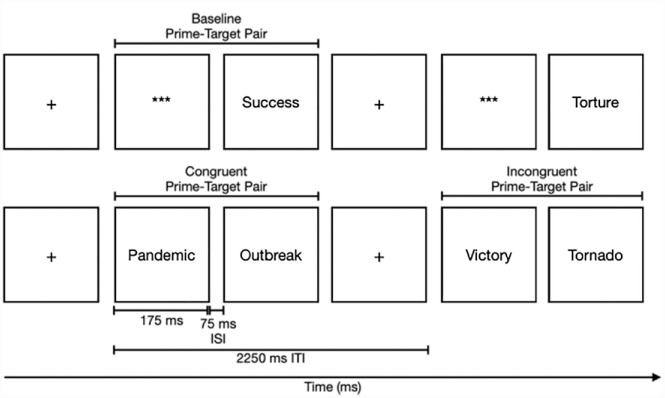
A schematic illustration of the presentation of affective word stimuli (adapted from Moro and Steeves, 2021).

Stimuli were made up of words from three affect categories: pleasant, unpleasant, and COVID-19. All stimuli were the same as those used in Moro and Steeves (2021). Briefly, on each trial the affective valence of the prime and the target was either congruent or incongruent. There were 60 congruent trials where the prime and target were both pleasant, both unpleasant, or both COVID-19 related, with an equal number of trials for each affective category. Sixty incongruent trials where the prime was paired with a target that was not from the same affect category as the prime were included. Furthermore, 33 baseline trials where each word from each affect category was presented as a target paired with a string of asterisks (***) were also included and randomly presented intermixed with the congruent and incongruent trials. Baseline trials were included for participants to be able to explicitly categorize each word presented during the experiment as ‘pleasant’ or ‘unpleasant’. There was a total of 153 trials that were each 2250ms in length, where the prime stimulus was presented for 175 ms followed by a 75 ms inter-stimulus interval and the target stimulus was presented for the remaining 2000ms or until the participant made a response. Figure 1 provides a schematic illustration of the stimuli presentation. See Moro and Steeves (2021) and Appendix A for more detailed report of the word stimuli utilized.

### Procedure

All procedures were the same as those previously used by Moro and Steeves (2021). Participants completed the entirety of this study online between August through December 2021 due to university restrictions to in-person participation because of the ongoing COVID-19 global pandemic. Qualtrics (Qualtrics, 2020) was used to administer the informed consent and demographic portion of this study and Inquisit was used for the affective priming portion. Following the completion of the informed consent and demographic section, participants were asked to complete the COVID-19 Pandemic Mental Health Questionnaire (CoPaQ) (Rek et al., 2021). The CoPaQ assesses COVID-19 contamination anxiety, countermeasure necessity and compliance, mental health impact, stressor impact, social media usage, interpersonal conflicts, paranoid ideations, institutional and political trust, conspiracy beliefs, and social cohesions (Rek et al., 2021). After completion of the questionnaire, participants proceeded with the affective priming experiment. Participants were asked to categorize affective target words into pleasant and unpleasant categories. Each participant completed 153 trials of randomly presented congruent and incongruent prime-target pairings, as well as baseline trials. Participants were instructed to respond as quickly and as accurately as possible by ignoring the first word (prime stimulus) and to classify the second word (target) as either pleasant or unpleasant using two corresponding buttons on their keyboard.

## Results

### COVID-19 attitudes

Measures of risk perception, necessary precaution, and adherence to COVID-19 safety measures were averaged based on a series of questions outlined under each topic in the CoPaQ questionnaire (Rek et al., 2021). All items were scored out of 4, where 0 was ranked lowest (‘not at all’) and 4 was ranked highest (‘very much’). A Greenhouse-Geisser corrected 3 × 2 repeated measures analysis of variance (ANOVA) was conducted to compare the overall rating for COVID-19 attitudes (risk perception, necessary precaution, and adherence to public health measures) for vaccine-hesitant participants compared to pro-vaccination participants. There was a significant main effect for COVID-19 attitude, *F*(1.65, 62.57) = 72.398, *p* < 0.001, *η*_*p*_^2^ = 0.656 and participant group, *F*(1, 38) = 5.689, *p* = 0.022, *η*_*p*_^2^ = 0.130. There was no significant interaction, *F*(1.65, 62.57) = 0.883, *p* = 0.400, *η*_*p*_^2^ = 0.023. Tukey corrected post hoc comparisons were conducted for the vaccine-hesitant participants that indicated significantly lower perception of risk compared to the perceived need for necessary precautions (*p* = 0.001) and perceived adherence to public health measures (*p* < 0.001), as well as, significantly lower perception of the needs for necessary precautions compared to perceived adherence to public health measures (*p* < 0.001). For pro-vaccination participants similar results were observed, indicating significantly lower perception of risk compared to the perceived need for necessary precautions (*p* = 0.006) and perceived adherence to public health measures (*p* < 0.001), as well as, significantly lower perception of the needs for necessary precautions compared to perceived adherence to public health measures (*p* = 0.006).

**Figure 2.**
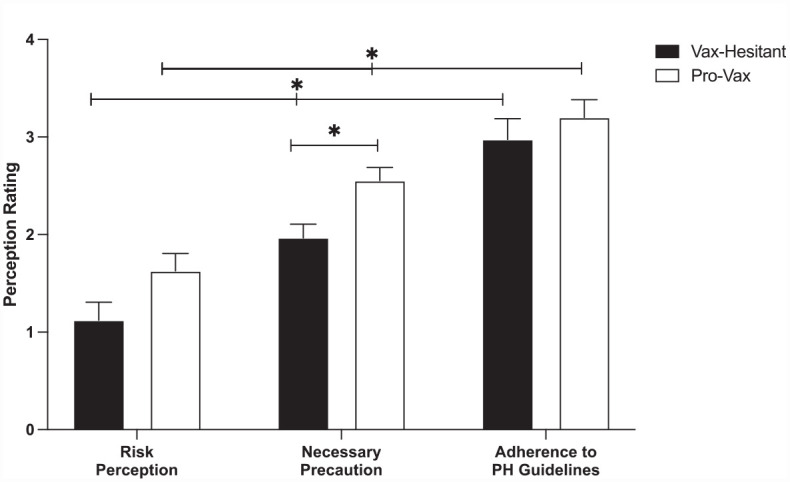
Self-reported rating on a 4-factor scale (where 4 indicates the greatest importance) for COVID-19 risk perception, perceived necessity for public health measures to mitigate COVID-19, and perception of an individual’s adherence to public health (PH) measures for vaccine-hesitant participants (black bars) and pro-vaccine participants (white bars).

Additionally, between groups Tukey corrected post hoc comparisons were conducted to examine whether there were group differences in the overall rating for COVID-19 attitudes (risk perception, necessary precaution, and adherence to public health measures). Pro-vaccination participants explicitly rated their perception for the necessity for public health precautions higher compared to vaccine hesitant participants (*p* = 0.048). There was no difference between pro-vaccine and vaccine-hesitant participants in their reported perception of risk (*p* = 0.382) and perceived adherence to public health measures (*p* = 0.965). Figure 2 plots the perceived risk, the perception of necessity for public health measures, and the perceived adherence to public health measures for the vaccine-hesitant and the pro-vaccine participants.

### Proportion of unpleasant responses

A 3 × 2 repeated measures ANOVA was conducted to compare the overall proportion of unpleasant responses for affective words (COVID-19 related, pleasant, and unpleasant) for vaccine-hesitant participants compared to pro-vaccination participants. There was a significant main effect for proportion of unpleasant responses, *F*(2, 76) = 3356.15, *p* < 0.001, *η*_*p*_^2^ = 0.989 and interaction, *F*(2, 76) = 7.58, *p* < 0.001, *η*_*p*_^2^ = 0.116. There was no significant main effect for participant group, *F*(1, 38) = 1.43, *p* = 0.240, *η*_*p*_^2^ = 0.036. Tukey corrected post hoc comparisons were conducted for the vaccine-hesitant participants that indicated significantly lower proportion of unpleasant responses for pleasant word stimuli compared to COVID-19 related word stimuli (*p* < 0.001) and unpleasant word stimuli (*p* < 0.001), as well as, significantly lower perception of unpleasant responses for COVID-19 related word stimuli compared to unpleasant word stimuli (*p* < 0.001). Despite a significant difference observed between the COVID-19 related word stimuli and the unpleasant word stimuli, it is important to note that COVID-19 related words were consistently rated as unpleasant (*M* = 0.90, SD = 0.08).

For pro-vaccination participants similar results were observed, indicating significantly lower proportion of unpleasant responses for pleasant word stimuli compared to COVID-19 related word stimuli (*p* < 0.001) and unpleasant word stimuli (*p* < 0.001). There was no difference in the proportion of unpleasant responses for COVID-19 related word stimuli compared to unpleasant word stimuli (*p* = 0.997).

**Figure 3.**
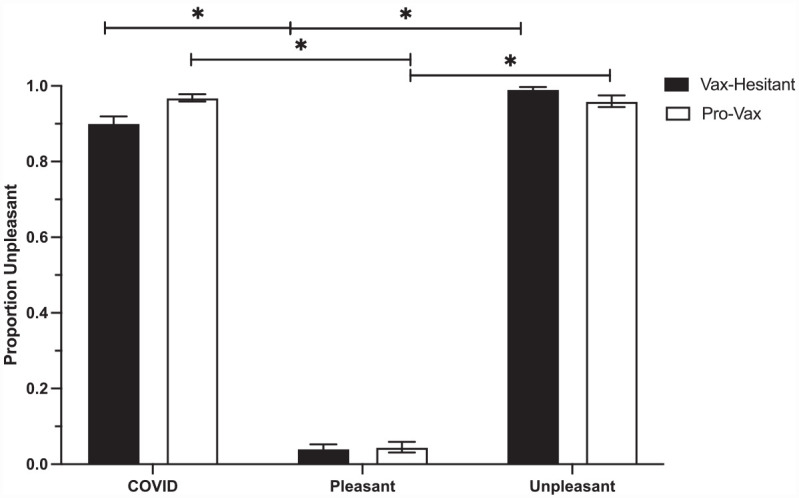
The proportion of unpleasant responses for each of the COVID-19, pleasant, and unpleasant word categories for vaccine-hesitant participants (black bars) and pro-vaccine participants (white bars). Both unpleasant and COVID-19 words were most often rated as unpleasant while pleasant words were rarely rated as unpleasant.

Additionally, between groups Tukey corrected post hoc comparisons were conducted to examine whether there were group differences in the overall rating for proportion of unpleasant responses for affective word categories between pro-vaccination and vaccine hesitant participants. Pro-vaccination participants rated the COVID-19 related word stimuli as unpleasant significantly more compared to vaccine-hesitant participants (*p* = 0.028). There was no difference between pro-vaccine and vaccine-hesitant participants in their rating of proportion unpleasant for unpleasant word stimuli (*p* = 0.412) and pleasant word stimuli (*p* = 1.000). Figure 3 plots the proportion unpleasant rated for each of the pleasant, unpleasant, and COVID-19 word categories for vaccine-hesitant and pro-vaccine participants.

### Reaction time

#### Baseline reaction time

**Figure 4.**
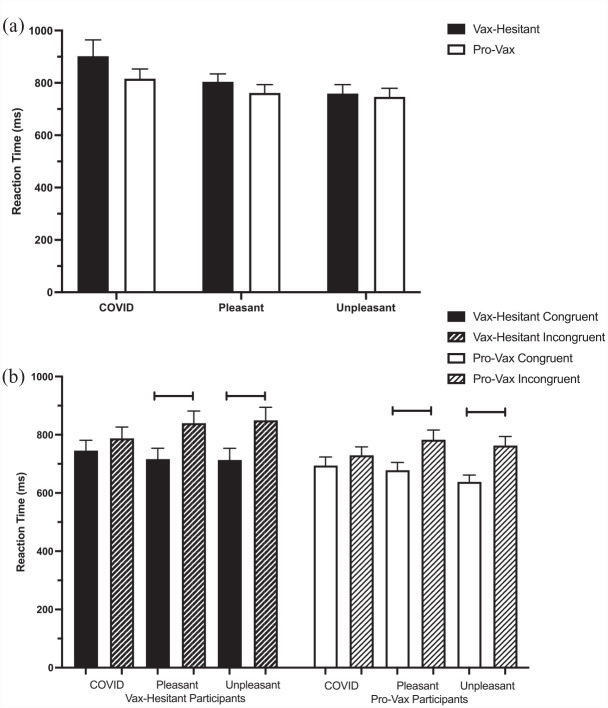
(a) The baseline reaction times for each of the COVID-19, pleasant, and unpleasant word categories for vaccine-hesitant (black) and pro-vaccine (white) participants. (b) The congruent (solid) and incongruent (striped) prime-target reaction times for each of the COVID-19 pleasant and unpleasant word categories for vaccine-hesitant (black) and pro-vaccine (white) participants. Affective priming was observed for pleasant and unpleasant affective word categories but not COVID-19 related words for both vaccine-hesitant and pro-vaccination groups.

A Greenhouse-Geisser corrected 3 × 2 repeated measures ANOVA was conducted to compare the baseline reaction times for affective word categories (COVID-19 related, pleasant, and unpleasant) for vaccine-hesitant participants compared to pro-vaccination participants. There was a significant main effect for baseline reaction times, *F*(1.66, 63.12) = 5.608, *p* = 0.005, *η*_*p*_^2^ = 0.129. There was no significant interaction, *F*(1.66, 63.12) = 0.645, *p* = 0.500, *η*_*p*_^2^ = 0.017 or main effect of participant group, *F*(1, 38) = 1.32, *p* = 0.259, *η*_*p*_^2^ = 0.033. Tukey corrected post hoc comparisons indicate no differences between participant groups and between reaction times for affective word categories for each group. Figure 4(a) plots the reaction time for each of the baseline pleasant, unpleasant, and COVID-19 word categories for vaccine-hesitant and pro-vaccine participants.

#### Congruent and incongruent reaction time

A Greenhouse-Geisser corrected 6 × 2 repeated measures ANOVA was conducted to compare the overall reaction times for congruent and incongruent affective word stimuli (COVID-19 related congruent words, COVID-19 related incongruent words, pleasant congruent words, pleasant incongruent words, unpleasant congruent words, and unpleasant incongruent words) for vaccine-hesitant participants compared to pro-vaccination participants. There was a significant main effect for reaction time, *F*(3.60, 136.99) = 18.44, *p* < 0.001, *η*_*p*_^2^ = 0.327. There was no significant main effect of participant group, *F*(1, 38) = 2.03, *p* = 0.163, *η*_*p*_^2^ = 0.051 or interaction, *F*(3.60, 136.99) = 0.435, *p* = 0.764, *η*_*p*_^2^ = 0.011. Tukey corrected post hoc comparisons were conducted for the vaccine-hesitant participants and indicated that congruent reaction times were significantly faster for pleasant words (*p* = 0.001) and unpleasant words (*p* = 0.013) compared to incongruent reaction times. Congruent reaction times for COVID-19 related words did not differ from incongruent reaction times for COVID-19 words (*p* = 0.691). These results indicate an affective priming effect for pleasant and unpleasant words but not COVID-19 related words. For pro-vaccination participants similar results were observed, indicating that congruent reaction times were significantly faster for pleasant words (*p* = 0.009) and unpleasant words (*p* = 0.030) compared to incongruent reaction times. Congruent reaction times for COVID-19 related words did not differ from incongruent reaction times for COVID-19 words (*p* = 0.865). Similar to vaccine-hesitant participants, these results indicate an affective priming effect for pleasant and unpleasant words but not COVID-19 related words.

Figure 4(b) plots the reaction time for each of the congruent and incongruent prime-target word pairs for the pleasant, unpleasant, and COVID-19 word categories for vaccine-hesitant and pro-vaccine participants.

## Discussion

The current study used affective priming as an indirect behavioural measure aimed at evaluating implicit COVID-19 attitudes in vaccine-hesitant and pro-vaccine participants. When asked explicitly, all participants reported a significantly lower perception of risk associated with contracting COVID-19 compared to their perceived necessity of COVID-19 precautionary measures and adherence to public health measures. Pro-vaccine participants rated their perception of the importance of necessary precautions to avoid contracting COVID-19 as higher compared to vaccine-hesitant participants. During baseline trials of our priming task, all participants rated COVID-19 affiliated words as unpleasant, similar to traditional unpleasant word stimuli. For both groups, baseline reaction times to COVID-19 words did not differ compared to baseline reaction times to both pleasant and unpleasant words indicating that word length and familiarity did not impact our results. Affective priming was observed for the pleasant and unpleasant prime conditions for both vaccine-hesitant and pro-vaccine participants. Affective priming was not observed for the COVID-19 prime condition for both vaccine-hesitant and pro-vaccine participants. Overall, these results provide further quantitative evidence that the affective priming paradigm is effective at measuring implicit attitudes towards COVID-19. Additionally, an attitude-behaviour discrepancy exists in both pro-vaccination and vaccine-hesitant demographic of the population.

Indirect implicit measures of attitude are important as they are less influenced by normative social demands and could potentially better predict behaviour in some circumstances (Greenwald and Banaji, 1995). Affective priming is frequently used as an indirect behavioural measure of attitude (Abbassi et al., 2019; Dovidio et al., 1997; Fazio et al., 1995; Greenwald and Farnham, 2000; Hermans et al., 2002; Lamote et al., 2004; Marsh et al., 2001; Spalding and Hardin, 1999; Teachman et al., 2001; Wittenbrink et al., 1997). Recently, it has been used to evaluate attitudes towards COVID-19 (Moro and Steeves, 2021). Explicitly, participants rated their overall risk perception associated with contracting COVID-19 significantly lower compared to their perception of necessary precautions and overall adherence to public health measures (Moro and Steeves, 2021). Implicitly, affective priming was not observed for COVID-19 affiliated prime-target word pairs when compared to pleasant and unpleasant words (Moro and Steeves, 2021). This initial study provided quantitative evidence that COVID-19 affiliated words do not invoke the same implicit attitude response as traditional pleasant and unpleasant word stimuli, possibly due to participants not internalizing their unpleasant attitude towards the COVID-19 word category. The current study replicated the previous results as the lack of affective priming observed in both participant groups indicates that they have not internalized their explicitly reported unpleasant attitude towards the COVID-19 related word category. In other words, while COVID-19 words were categorized as unpleasant they did not generate a faster response when paired congruently. This is exemplified by our baseline evaluations where COVID-19 related words were strongly associated with unpleasant attitudes but were not reflective of the participants’ implicit affective priming performance for traditional pleasant and unpleasant word pairs. As implicit attitudes are often used as a good predictor for future behaviour, our current results indicate that the attitude-behaviour discrepancy previously observed is replicated when specifically controlling for those who identify as being vaccine-hesitant as opposed to pro-vaccine. The attitude-behaviour discrepancy is illustrated by the COVID-19 attitudes measured for each participant group. When explicitly asked, both vaccine-hesitant and pro-vaccine participants reported a low perception of risk compared to their perceived necessity of public health measures and adherence to public health measures. The lower perception of risk is reflective of the participants’ implicit attitude towards COVID-19, demonstrated through a lack of affective priming, indicating that they are less fearful of the COVID-19 virus but not aligned with the explicit rating of COVID-19 related words as unpleasant. This is further exemplified by the higher explicit rating for adherence to public health guidelines. If implicit attitudes and explicit rating of risk perception is low, people are not likely to modify their behaviour to accommodate public health guidelines to reduce the spread of the COVID-19 virus.

There has consistently been a reported relationship between risk perception and behaviour, specifically with vaccination willingness, where increased risk perception is associated with higher vaccination behaviour (Brewer et al., 2007). This relationship between risk perception and health behaviour is also applicable to the ongoing COVID-19 pandemic (Harper et al., 2021). In our current study, the pro-vaccine group rated the necessity of public health measures significantly higher compared to the vaccine-hesitant group which is aligned with their willingness to be vaccinated. Furthermore, this might be an indication that the low perception of risk between the groups might occur through two separate pathways for each group. We can speculate that this difference might be a reflection of the vaccination status between the groups. The low perceived risk reported for pro-vaccine participants might be a result of their vaccination status as the vaccine is offering them protection against severe illness due to COVID-19. Whereas, those who are not vaccinated have reported a lower necessity for public health measures. It has been shown that vaccine hesitancy has been associated with lower perceived susceptibility to COVID-19, higher perceived barriers, and lower perceived benefits (Du et al., 2021; Kelly et al., 2021; Thunström et al., 2021; Viswanath et al., 2021). Despite vaccine-hesitant participants’ lower rating of necessity for public health measures both groups rated their adherence to public health measures as high. In the case of vaccine-hesitant participants this may be due to the vaccine mandates enforced by workplaces as a requirement for some of our vaccine-hesitant participants to receive a vaccine in order to return to work, regardless of their personal beliefs. Additionally, the strong reports of adherence to public health measures may be indicative of the influence of normative pressures rather than reflective of explicit or implicit COVID-19 risk perception. It is likely that both groups are experiencing an attitude-behaviour discrepancy based on the low perception of risk due to COVID-19 reported, the perception of strong adherence to public health measures for both groups, and explicit rating of COVID-19 affiliated words as unpleasant indicating that they have not internalized the association between COVID-19 affiliated words as being unpleasant but perhaps each for different reasons.

The current study indicates that affective priming can be used to investigate implicit attitudes towards the COVID-19 pandemic and may help to explain why some demographic groups might be more willing to adhere to public health measures compared to others. A limitation of our current study is a small sample size of 40 participants, just under the recommended sample size of 44 participants based on our a-priori power analysis. Despite results obtaining small to medium effect sizes, future large-scale investigations across a wider age range of participants, population demographics, education level, verbal intelligence, and geographical locations would expand the current results. Furthermore, the mean age of our vaccine-hesitant participants is (55 years) older compared to our pro-vaccine participants (38 years). Future studies investigating the impact of participant age on vaccination hesitancy would contribute to interpreting the current results. In general, it has been shown that young people are not motivated by fear-related behaviours due to decreased severity of COVID-19-related illness in younger age groups (Bai et al., 2020; Lai et al., 2020). If this were the case in our participant group, we would expect the average age of vaccine-hesitant participants to be lower. Overall, our current results indicate that regardless of age, the attitude-behaviour discrepancy is still observed. Additionally, it is possible that willingness to be vaccinated is a contributing factor to the evidence that participants demonstrate decreased risk perception towards COVID-19.

In conclusion, this study demonstrated an attitude-behaviour discrepancy in both vaccine-hesitant and pro-vaccination participants where they explicitly rated COVID-19 related words as unpleasant, but implicitly did not demonstrate affective priming. This may contribute towards decreased fear-related behaviours and increased incidence of risky-behaviour facilitating the spread of the virus. Overall, this study contributes to the growing body of research exploring why some individuals across communities might be more or less willing to engage in precautionary behaviours outlined by their public health agencies to mitigate the spread of COVID-19.

## Research Data

sj-docx-1-hpq-10.1177_13591053231176261 – Supplemental material for Assessment of implicit COVID-19 attitudes using affective priming for pro-vaccine and vaccine-hesitant individuals Supplemental material, sj-docx-1-hpq-10.1177_13591053231176261 for Assessment of implicit COVID-19 attitudes using affective priming for pro-vaccine and vaccine-hesitant individuals by Stefania S Moro and Jennifer K E Steeves in Journal of Health Psychology

sj-pdf-2-hpq-10.1177_13591053231176261 – Supplemental material for Assessment of implicit COVID-19 attitudes using affective priming for pro-vaccine and vaccine-hesitant individuals Supplemental material, sj-pdf-2-hpq-10.1177_13591053231176261 for Assessment of implicit COVID-19 attitudes using affective priming for pro-vaccine and vaccine-hesitant individuals by Stefania S Moro and Jennifer K E Steeves in Journal of Health Psychology

sj-pdf-3-hpq-10.1177_13591053231176261 – Supplemental material for Assessment of implicit COVID-19 attitudes using affective priming for pro-vaccine and vaccine-hesitant individuals Supplemental material, sj-pdf-3-hpq-10.1177_13591053231176261 for Assessment of implicit COVID-19 attitudes using affective priming for pro-vaccine and vaccine-hesitant individuals by Stefania S Moro and Jennifer K E Steeves in Journal of Health Psychology

sj-xlsx-4-hpq-10.1177_13591053231176261 – Supplemental material for Assessment of implicit COVID-19 attitudes using affective priming for pro-vaccine and vaccine-hesitant individuals Supplemental material, sj-xlsx-4-hpq-10.1177_13591053231176261 for Assessment of implicit COVID-19 attitudes using affective priming for pro-vaccine and vaccine-hesitant individuals by Stefania S Moro and Jennifer K E Steeves in Journal of Health Psychology This article is distributed under the terms of the Creative Commons Attribution 4.0 License ( http://www.creativecommons.org/licenses/by/4.0/) which permits any use, reproduction and distribution of the work without further permission provided the original work is attributed as specified on the SAGE and Open Access pages ( https://us.sagepub.com/en-us/nam/open-access-at-sage).

## Appendix

**Appendix A.** Affective priming words by category.

Pleasant primes	Pleasant targets	Unpleasant primes	Unpleasant targets	COVID-19 primes	Covid-19 targets
Triumph	Bouquet	Horror	Torture	Pandemic	Outbreak
Delight	Success	Traitor	Quarrel	Mask	Cases
Glory	Diamond	Hatred	Tornado	Lockdown	Isolation
Blossom	Passion	Misery	Deceit	Virus	Sanitizer
Victory	Fantasy	Prison	Robber	Quarantine	Fever
Miracle		Assault		Spread	
