# Supplementary material for: Assessment of implicit COVID-19 attitudes using affective priming for pro-vaccine and vaccine-hesitant individuals
Source: J Health Psychol. 2023 Jun 2;28(14):1331–44. doi: 10.1177/13591053231176261 (PMC10240302; doi:10.1177/13591053231176261)
Supplement: sj-docx-1-hpq-10.1177_13591053231176261 – Supplemental material for Assessment of implicit COVID-19 attitudes using affective priming for pro-vaccine and vaccine-hesitant individuals [file sj-docx-1-hpq-10.1177_13591053231176261.docx]

An implicit measure of COVID-19 attitudes using affective priming for pro-vaccine and vaccine-hesitant individuals

**Data Set Explanatory Memo**

This dataset contains reaction time and proportion correct data for an affective priming task where participants were asked to respond to pleasant, unpleasant, and COVID-19 related words. 40 participants who are self-described native English speakers and self-identify as vaccine-hesitant or pro-vaccination are included.

Variables:

1. **Age:** Age of participants (years)
2. **Sex:** Self-identified biological sex of participants (male/female)
3. **Vax Status:** Self-identified vaccination status of participants (vaccine hesitant/ pro-vaccination)
4. **CoPaQ Score:** Self-reported scores from the COVID-19 Pandemic Mental Health Questionnaire (Rek et al., 2020; Rek et al., under review) on 3 items: Risk Perception, Necessary Precaution, Adherence to Public Health (PH) Measures.
5. **Reaction Time:** Reaction time (ms) separated by baseline measures (COVID, Pleasant, Unpleasant) and Prime-Target Pairs (COVID Congruent, COVID Incongruent, Pleasant Congruent, Pleasant Incongruent, Unpleasant Congruent, Unpleasant Incongruent).
6. **Proportion Unpleasant:** Proportion unpleasant responses for Baseline affective measures (COVID, Pleasant, Unpleasant).
7. **Proportion Correct:** Proportion correct for Prime-Target Pairs (COVID Congruent, COVID Incongruent, Pleasant Congruent, Pleasant Incongruent, Unpleasant Congruent, Unpleasant Incongruent).

Data Analysis Software:

1. jamovi (version 2.3.18.0)
