# Supplementary material for: Assessment of implicit COVID-19 attitudes using affective priming for pro-vaccine and vaccine-hesitant individuals
Source: J Health Psychol. 2023 Jun 2;28(14):1331–44. doi: 10.1177/13591053231176261 (PMC10240302; doi:10.1177/13591053231176261)
Supplement: sj-pdf-3-hpq-10.1177_13591053231176261 – Supplemental material for Assessment of implicit COVID-19 attitudes using affective priming for pro-vaccine and vaccine-hesitant individuals [file sj-pdf-3-hpq-10.1177_13591053231176261.pdf]

Results

Repeated Measures ANOVA

Within Subjects Effects

	Sphericity Correction	Sum of Squares	df	Mean Square	F	p	η^2_p
COVID Perception	None	58.709	2	29.355	72.398	< .001	0.656
	Greenhouse-Geisser	58.709	1.65	35.658	72.398	< .001	0.656
COVID Perception ✻ Participant Group (2)	None	0.716	2	0.358	0.883	0.418	0.023
	Greenhouse-Geisser	0.716	1.65	0.435	0.883	0.400	0.023
Residual	None	30.815	76	0.405			
	Greenhouse-Geisser	30.815	62.57	0.493			

Note. Type 3 Sums of Squares

[3]

Between Subjects Effects

	Sum of Squares	df	Mean Square	F	p	η^2_p
Participant Group (2)	5.77	1	5.77	5.68	0.022	0.130
Residual	38.62	38	1.02			

Note. Type 3 Sums of Squares

Assumptions

Tests of Sphericity

	Mauchly's W	p	Greenhouse-Geisser ε	Huynh-Feldt ε
COVID Perception	0.785	0.011	0.823	0.856

Homogeneity of Variances Test (Levene's)

	F	df1	df2	p
Risk Perception	0.42888	1	38	0.516
Necessity of PH	0.20678	1	38	0.652
Adherence to PH	0.00637	1	38	0.937

Post Hoc Tests

Post Hoc Comparisons - COVID Perception

Comparison								
COVID Perception		COVID Perception	Mean Difference	SE	df	t	p_{tukey}	$p_{bonferroni}$
Risk Perception	-	Necessity of PH	-0.885	0.130	38.0	-6.83	<.001	<.001
	-	Adherence to PH	-1.713	0.172	38.0	-9.96	<.001	<.001
Necessity of PH	-	Adherence to PH	-0.828	0.120	38.0	-6.88	<.001	<.001

Post Hoc Comparisons - Participant Group (2)

Comparison								
Participant Group (2)		Participant Group (2)	Mean Difference	SE	df	t	p_{tukey}	$p_{bonferroni}$
ProVax	-	AntiVax	0.438	0.184	38.0	2.38	0.022	0.022

Post Hoc Comparisons - COVID Perception ✻ Participant Group (2)

Comparison										
COVID Perception	Participant Group (2)		COVID Perception	Participant Group (2)	Mean Difference	SE	df	t	p_{tukey}	$p_{bonferroni}$
Risk Perception	ProVax	-	Risk Perception	AntiVax	0.505	0.257	38.0	1.963	0.382	0.855
		-	Necessity of PH	ProVax	-0.926	0.183	38.0	-5.052	<.001	<.001
		-	Necessity of PH	AntiVax	-0.340	0.228	38.0	-1.491	0.672	1.000
		-	Adherence to PH	ProVax	-1.573	0.243	38.0	-6.468	<.001	<.001
		-	Adherence to PH	AntiVax	-1.348	0.269	38.0	-5.007	<.001	<.001
	AntiVax	-	Necessity of PH	ProVax	-1.431	0.228	38.0	-6.275	<.001	<.001
		-	Necessity of PH	AntiVax	-0.845	0.183	38.0	-4.613	<.001	<.001
		-	Adherence to PH	ProVax	-2.078	0.269	38.0	-7.719	<.001	<.001
		-	Adherence to PH	AntiVax	-1.853	0.243	38.0	-7.619	<.001	<.001
Necessity of PH	ProVax	-	Necessity of PH	AntiVax	0.586	0.194	38.0	3.013	0.048	0.069
		-	Adherence to PH	ProVax	-0.647	0.170	38.0	-3.807	0.006	0.007
		-	Adherence to PH	AntiVax	-0.422	0.241	38.0	-1.750	0.509	1.000
	AntiVax	-	Adherence to PH	ProVax	-1.233	0.241	38.0	-5.108	<.001	<.001
		-	Adherence to PH	AntiVax	-1.008	0.170	38.0	-5.926	<.001	<.001
Adherence to PH	ProVax	-	Adherence to PH	AntiVax	0.225	0.281	38.0	0.802	0.965	1.000

[4]

Repeated Measures ANOVA

Within Subjects Effects

	Sphericity Correction	Sum of Squares	df	Mean Square	F	p	η^2_p
Proportion of Unpleasant	None	22.2248	2	11.11240	3356.15	< .001	0.989
	Greenhouse-Geisser	22.2248	1.93	11.49072	3356.15	< .001	0.989
Proportion of Unpleasant ✻ Participant Group (2)	None	0.0502	2	0.02511	7.58	< .001	0.166
	Greenhouse-Geisser	0.0502	1.93	0.02596	7.58	0.001	0.166
Residual	None	0.2516	76	0.00331			
	Greenhouse-Geisser	0.2516	73.50	0.00342			

Note. Type 3 Sums of Squares

[3]

Between Subjects Effects

	Sum of Squares	df	Mean Square	F	p	η^2_p
Participant Group (2)	0.00547	1	0.00547	1.43	0.240	0.036
Residual	0.14566	38	0.00383			

Note. Type 3 Sums of Squares

Assumptions

Tests of Sphericity

	Mauchly's W	p	Greenhouse-Geisser ε	Huynh-Feldt ε
Proportion of Unpleasant	0.966	0.527	0.967	1.00

Homogeneity of Variances Test (Levene's)

	F	df1	df2	p
COVID Prop Unpleasant	5.177	1	38	0.029
Pleasant Prop Unpleasant	0.469	1	38	0.498
Unpleasant Prop Unpleasant	18.480	1	38	< .001

Post Hoc Tests

Post Hoc Comparisons - Proportion of Unpleasant

Comparison								
Proportion of Unpleasant		Proportion of Unpleasant	Mean Difference	SE	df	t	p_{tukey}	$p_{bonferroni}$
COVID	-	Pleasant	0.8920	0.0130	38.0	68.85	< .001	< .001
	-	Unpleasant	-0.0405	0.0138	38.0	-2.93	0.015	0.017
Pleasant	-	Unpleasant	-0.9325	0.0117	38.0	-79.37	< .001	< .001

Post Hoc Comparisons - Participant Group (2)

Comparison								
Participant Group (2)		Participant Group (2)	Mean Difference	SE	df	t	p_{tukey}	$p_{bonferroni}$
ProVax	-	AntiVax	0.0135	0.0113	38.0	1.19	0.240	0.240

Post Hoc Comparisons - Proportion of Unpleasant ✻ Participant Group (2)

Comparison											
Proportion of Unpleasant	Participant Group (2)		Proportion of Unpleasant	Participant Group (2)	Mean Difference	SE	df	t	p_{tukey}	$p_{bonferroni}$
COVID	ProVax	-	COVID	AntiVax	0.06750	0.0208	38.0	3.241	0.028	0.037
		-	Pleasant	ProVax	0.92350	0.0183	38.0	50.402	<.001	<.001
		-	Pleasant	AntiVax	0.92800	0.0197	38.0	47.180	<.001	<.001
		-	Unpleasant	ProVax	0.00900	0.0195	38.0	0.461	0.997	1.000
		-	Unpleasant	AntiVax	-0.02250	0.0188	38.0	-1.198	0.835	1.000
	AntiVax	-	Pleasant	ProVax	0.85600	0.0197	38.0	43.520	<.001	<.001
		-	Pleasant	AntiVax	0.86050	0.0183	38.0	46.963	<.001	<.001
		-	Unpleasant	ProVax	-0.05850	0.0188	38.0	-3.115	0.038	0.052
		-	Unpleasant	AntiVax	-0.09000	0.0195	38.0	-4.607	<.001	<.001
Pleasant	ProVax	-	Pleasant	AntiVax	0.00450	0.0184	38.0	0.244	1.000	1.000
		-	Unpleasant	ProVax	-0.91450	0.0166	38.0	-55.042	<.001	<.001
		-	Unpleasant	AntiVax	-0.94600	0.0175	38.0	-54.090	<.001	<.001
	AntiVax	-	Unpleasant	ProVax	-0.91900	0.0175	38.0	-52.546	<.001	<.001
		-	Unpleasant	AntiVax	-0.95050	0.0166	38.0	-57.209	<.001	<.001
Unpleasant	ProVax	-	Unpleasant	AntiVax	-0.03150	0.0165	38.0	-1.911	0.412	0.954

[4]

Repeated Measures ANOVA

Within Subjects Effects

	Sphericity Correction	Sum of Squares	df	Mean Square	F	p	η^2_p
Baseline RT	None	238219	2	119110	5.608	0.005	0.129
	Greenhouse-Geisser	238219	1.66	143412	5.608	0.009	0.129
Baseline RT ✻ Participant Group (2)	None	27412	2	13706	0.645	0.527	0.017
	Greenhouse-Geisser	27412	1.66	16503	0.645	0.500	0.017
Residual	None	1.61e+6	76	21240			
	Greenhouse-Geisser	1.61e+6	63.12	25574			

Note. Type 3 Sums of Squares

[3]

Between Subjects Effects

	Sum of Squares	df	Mean Square	F	p	η^2_p
Participant Group (2)	67000	1	67000	1.32	0.259	0.033
Residual	1.93e+6	38	50917			

Note. Type 3 Sums of Squares

Assumptions

Tests of Sphericity

	Mauchly's W	p	Greenhouse-Geisser ε	Huynh-Feldt ε
Baseline RT	0.796	0.015	0.831	0.864

Homogeneity of Variances Test (Levene's)

	F	df1	df2	p
Baseline COVID RT	3.3927	1	38	0.073
Baseline Pleasant RT	0.0106	1	38	0.919
Baseline Unpleasant RT	0.2053	1	38	0.653

Post Hoc Tests

Post Hoc Comparisons - Baseline RT

Comparison								
Baseline RT		Baseline RT	Mean Difference	SE	df	t	p_{tukey}	$p_{bonferroni}$
COVID	-	Pleasant	76.0	33.3	38.0	2.28	0.070	0.084
	-	Unpleasant	105.8	38.1	38.0	2.78	0.023	0.025
Pleasant	-	Unpleasant	29.8	25.0	38.0	1.19	0.465	0.721

Post Hoc Comparisons - Participant Group (2)

Comparison								
Participant Group (2)		Participant Group (2)	Mean Difference	SE	df	t	p_{tukey}	$p_{bonferroni}$
ProVax	-	AntiVax	-47.3	41.2	38.0	-1.15	0.259	0.259

Post Hoc Comparisons - Baseline RT ✻ Participant Group (2)

Comparison										
**Baseline RT**	**Participant Group (2)**		**Baseline RT**	**Participant Group (2)**	**Mean Difference**	**SE**	**df**	**t**	**p_{tukey}**	**$p_{bonferroni}$**
COVID	ProVax	-	COVID	AntiVax	-86.13	72.5	38.0	-1.1885	0.840	1.000
		-	Pleasant	ProVax	54.54	47.1	38.0	1.1586	0.853	1.000
		-	Pleasant	AntiVax	11.32	59.7	38.0	0.1896	1.000	1.000
		-	Unpleasant	ProVax	68.98	53.9	38.0	1.2798	0.794	1.000
		-	Unpleasant	AntiVax	56.56	61.1	38.0	0.9260	0.937	1.000
	AntiVax	-	Pleasant	ProVax	140.68	59.7	38.0	2.3557	0.198	0.356
		-	Pleasant	AntiVax	97.45	47.1	38.0	2.0701	0.324	0.679
		-	Unpleasant	ProVax	155.11	61.1	38.0	2.5397	0.138	0.230
		-	Unpleasant	AntiVax	142.69	53.9	38.0	2.6475	0.110	0.176
Pleasant	ProVax	-	Pleasant	AntiVax	-43.22	43.4	38.0	-0.9968	0.916	1.000
		-	Unpleasant	ProVax	14.43	35.4	38.0	0.4081	0.998	1.000
		-	Unpleasant	AntiVax	2.01	45.2	38.0	0.0445	1.000	1.000
	AntiVax	-	Unpleasant	ProVax	57.65	45.2	38.0	1.2753	0.796	1.000
		-	Unpleasant	AntiVax	45.23	35.4	38.0	1.2790	0.794	1.000
Unpleasant	ProVax	-	Unpleasant	AntiVax	-12.42	47.0	38.0	-0.2643	1.000	1.000

[4]

Repeated Measures ANOVA

Within Subjects Effects

	Sphericity Correction	Sum of Squares	df	Mean Square	F	p	η^2_p
Reaction Time	None	640541	5	128108	18.444	< .001	0.327
	Greenhouse-Geisser	640541	3.60	177686	18.444	< .001	0.327
Reaction Time ✻ Participant Group (2)	None	15102	5	3020	0.435	0.824	0.011
	Greenhouse-Geisser	15102	3.60	4189	0.435	0.764	0.011
Residual	None	1.32e+6	190	6946			
	Greenhouse-Geisser	1.32e+6	136.99	9634			

Note. Type 3 Sums of Squares

[3]

Between Subjects Effects

	Sum of Squares	df	Mean Square	F	p	η^2_p
Participant Group (2)	223781	1	223781	2.03	0.163	0.051
Residual	4.20e+6	38	110424			

Note. Type 3 Sums of Squares

Assumptions

Tests of Sphericity

	Mauchly's W	p	Greenhouse-Geisser ε	Huynh-Feldt ε
Reaction Time	0.389	0.002	0.721	0.806

Homogeneity of Variances Test (Levene's)

	F	df1	df2	p
COVID Congruent	0.418	1	38	0.522
COVID Incongruent	3.390	1	38	0.073
Pleasant Congruent	4.068	1	38	0.051
Pleasant Incongruent	1.415	1	38	0.242
Unpleasant Congruent	8.957	1	38	0.005
Unpleasant Incongruent	6.053	1	38	0.019

Post Hoc Tests

Post Hoc Comparisons - Reaction Time

Comparison								
Reaction Time		Reaction Time	Mean Difference	SE	df	t	p_{tukey}	$p_{bonferroni}$
Covid Congruent	-	Covid Incongruent	-38.42	14.8	38.0	-2.604	0.121	0.196
	-	Pleasant Congruent	22.97	13.0	38.0	1.769	0.497	1.000
	-	Pleasant Incongruent	-91.32	16.7	38.0	-5.459	< .001	< .001
	-	Unpleasant Congruent	44.14	15.8	38.0	2.796	0.080	0.121
	-	Unpleasant Incongruent	-86.06	23.7	38.0	-3.624	0.010	0.013
Covid Incongruent	-	Pleasant Congruent	61.39	13.0	38.0	4.729	< .001	< .001
	-	Pleasant Incongruent	-52.91	17.9	38.0	-2.954	0.056	0.080
	-	Unpleasant Congruent	82.56	16.9	38.0	4.886	< .001	< .001
	-	Unpleasant Incongruent	-47.64	20.6	38.0	-2.311	0.215	0.395
Pleasant Congruent	-	Pleasant Incongruent	-114.30	17.9	38.0	-6.386	< .001	< .001
	-	Unpleasant Congruent	21.17	14.9	38.0	1.419	0.715	1.000
	-	Unpleasant Incongruent	-109.03	21.4	38.0	-5.087	< .001	< .001
Pleasant Incongruent	-	Unpleasant Congruent	135.46	22.3	38.0	6.061	< .001	< .001
	-	Unpleasant Incongruent	5.27	21.4	38.0	0.247	1.000	1.000
Unpleasant Congruent	-	Unpleasant Incongruent	-130.20	23.9	38.0	-5.439	< .001	< .001

Post Hoc Comparisons - Participant Group (2)

Comparison								
Participant Group (2)		Participant Group (2)	Mean Difference	SE	df	t	p_{tukey}	$p_{bonferroni}$
ProVax	-	AntiVax	-61.1	42.9	38.0	-1.42	0.163	0.163

Post Hoc Comparisons - Reaction Time ✻ Participant Group (2)

Comparison										
Reaction Time	Participant Group (2)		Reaction Time	Participant Group (2)	Mean Difference	SE	df	t	p_{tukey}	$p_{bonferroni}$
Covid Congruent	ProVax	-	Covid Congruent	AntiVax	-51.37	45.6	38.0	-1.1258	0.991	1.000
		-	Covid Incongruent	ProVax	-35.11	20.9	38.0	-1.6827	0.865	1.000
		-	Covid Incongruent	AntiVax	-93.09	47.0	38.0	-1.9811	0.702	1.000
		-	Pleasant Congruent	ProVax	16.30	18.4	38.0	0.8875	0.999	1.000
		-	Pleasant Congruent	AntiVax	-21.72	45.9	38.0	-0.4732	1.000	1.000
		-	Pleasant Incongruent	ProVax	-88.52	23.7	38.0	-3.7418	0.026	0.040
		-	Pleasant Incongruent	AntiVax	-145.50	49.5	38.0	-2.9367	0.169	0.370
		-	Unpleasant Congruent	ProVax	56.21	22.3	38.0	2.5180	0.360	1.000
		-	Unpleasant Congruent	AntiVax	-19.30	45.8	38.0	-0.4212	1.000	1.000
		-	Unpleasant Incongruent	ProVax	-68.46	33.6	38.0	-2.0386	0.666	1.000
		-	Unpleasant Incongruent	AntiVax	-155.02	50.5	38.0	-3.0689	0.128	0.261
	AntiVax	-	Covid Incongruent	ProVax	16.26	47.0	38.0	0.3460	1.000	1.000
		-	Covid Incongruent	AntiVax	-41.72	20.9	38.0	-1.9996	0.691	1.000
		-	Pleasant Congruent	ProVax	67.67	45.9	38.0	1.4742	0.939	1.000
		-	Pleasant Congruent	AntiVax	29.65	18.4	38.0	1.6147	0.893	1.000
		-	Pleasant Incongruent	ProVax	-37.15	49.5	38.0	-0.7498	1.000	1.000
		-	Pleasant Incongruent	AntiVax	-94.13	23.7	38.0	-3.9790	0.014	0.020
		-	Unpleasant Congruent	ProVax	107.58	45.8	38.0	2.3477	0.463	1.000
		-	Unpleasant Congruent	AntiVax	32.07	22.3	38.0	1.4364	0.948	1.000
		-	Unpleasant Incongruent	ProVax	-17.09	50.5	38.0	-0.3384	1.000	1.000
		-	Unpleasant Incongruent	AntiVax	-103.65	33.6	38.0	-3.0863	0.123	0.249
Covid Incongruent	ProVax	-	Covid Incongruent	AntiVax	-57.98	48.3	38.0	-1.2000	0.986	1.000
		-	Pleasant Congruent	ProVax	51.41	18.4	38.0	2.8003	0.220	0.527
		-	Pleasant Congruent	AntiVax	13.39	47.3	38.0	0.2834	1.000	1.000
		-	Pleasant Incongruent	ProVax	-53.41	25.3	38.0	-2.1085	0.620	1.000
		-	Pleasant Incongruent	AntiVax	-110.39	50.8	38.0	-2.1728	0.578	1.000
		-	Unpleasant Congruent	ProVax	91.33	23.9	38.0	3.8220	0.021	0.031
		-	Unpleasant Congruent	AntiVax	15.81	47.2	38.0	0.3350	1.000	1.000
		-	Unpleasant Incongruent	ProVax	-33.35	29.2	38.0	-1.1440	0.990	1.000

Post Hoc Comparisons - Reaction Time ✻ Participant Group (2)

Comparison									
Reaction Time	Participant Group (2)		Reaction Time	Participant Group (2)	Mean Difference	SE	df	t	p_{tukey}	$p_{bonferroni}$
		-	Unpleasant Incongruent	AntiVax	-119.91	51.7	38.0	-2.3172	0.483	1.000
	AntiVax	-	Pleasant Congruent	ProVax	109.39	47.3	38.0	2.3148	0.484	1.000
		-	Pleasant Congruent	AntiVax	71.38	18.4	38.0	3.8878	0.018	0.026
		-	Pleasant Incongruent	ProVax	4.58	50.8	38.0	0.0901	1.000	1.000
		-	Pleasant Incongruent	AntiVax	-52.41	25.3	38.0	-2.0691	0.646	1.000
		-	Unpleasant Congruent	ProVax	149.31	47.2	38.0	3.1644	0.104	0.202
		-	Unpleasant Congruent	AntiVax	73.79	23.9	38.0	3.0881	0.123	0.248
		-	Unpleasant Incongruent	ProVax	24.63	51.7	38.0	0.4760	1.000	1.000
		-	Unpleasant Incongruent	AntiVax	-61.93	29.2	38.0	-2.1242	0.610	1.000
Pleasant Congruent	ProVax	-	Pleasant Congruent	AntiVax	-38.02	46.2	38.0	-0.8233	0.999	1.000
		-	Pleasant Incongruent	ProVax	-104.82	25.3	38.0	-4.1408	0.009	0.012
		-	Pleasant Incongruent	AntiVax	-161.80	49.8	38.0	-3.2491	0.086	0.160
		-	Unpleasant Congruent	ProVax	39.92	21.1	38.0	1.8929	0.756	1.000
		-	Unpleasant Congruent	AntiVax	-35.60	46.1	38.0	-0.7723	1.000	1.000
		-	Unpleasant Incongruent	ProVax	-84.76	30.3	38.0	-2.7964	0.222	0.532
		-	Unpleasant Incongruent	AntiVax	-171.32	50.8	38.0	-3.3751	0.064	0.113
	AntiVax	-	Pleasant Incongruent	ProVax	-66.80	49.8	38.0	-1.3414	0.968	1.000
		-	Pleasant Incongruent	AntiVax	-123.78	25.3	38.0	-4.8900	0.001	0.001
		-	Unpleasant Congruent	ProVax	77.93	46.1	38.0	1.6906	0.861	1.000
		-	Unpleasant Congruent	AntiVax	2.41	21.1	38.0	0.1145	1.000	1.000
		-	Unpleasant Incongruent	ProVax	-46.74	50.8	38.0	-0.9209	0.998	1.000
		-	Unpleasant Incongruent	AntiVax	-133.30	30.3	38.0	-4.3979	0.004	0.006
Pleasant Incongruent	ProVax	-	Pleasant Incongruent	AntiVax	-56.98	53.2	38.0	-1.0716	0.994	1.000
		-	Unpleasant Congruent	ProVax	144.73	31.6	38.0	4.5793	0.003	0.003
		-	Unpleasant Congruent	AntiVax	69.21	49.7	38.0	1.3919	0.958	1.000
		-	Unpleasant Incongruent	ProVax	20.06	30.2	38.0	0.6642	1.000	1.000
		-	Unpleasant Incongruent	AntiVax	-66.50	54.1	38.0	-1.2298	0.983	1.000
	AntiVax	-	Unpleasant Congruent	ProVax	201.71	49.7	38.0	4.0564	0.011	0.016
		-	Unpleasant Congruent	AntiVax	126.20	31.6	38.0	3.9929	0.013	0.019

Post Hoc Comparisons - Reaction Time ✻ Participant Group (2)

Comparison										
Reaction Time	Participant Group (2)		Reaction Time	Participant Group (2)	Mean Difference	SE	df	t	p_{tukey}	$p_{bonferroni}$
Unpleasant Congruent	ProVax	-	Unpleasant Incongruent	ProVax	77.04	54.1	38.0	1.4246	0.951	1.000
		-	Unpleasant Incongruent	AntiVax	-9.52	30.2	38.0	-0.3153	1.000	1.000
		-	Unpleasant Congruent	AntiVax	-75.52	46.0	38.0	-1.6409	0.883	1.000
		-	Unpleasant Incongruent	ProVax	-124.68	33.9	38.0	-3.6831	0.030	0.047
		-	Unpleasant Incongruent	AntiVax	-211.23	50.7	38.0	-4.1671	0.008	0.011
	AntiVax	-	Unpleasant Incongruent	ProVax	-49.16	50.7	38.0	-0.9698	0.998	1.000
		-	Unpleasant Incongruent	AntiVax	-135.72	33.9	38.0	-4.0092	0.013	0.018
Unpleasant Incongruent	ProVax	-	Unpleasant Incongruent	AntiVax	-86.56	55.0	38.0	-1.5748	0.908	1.000

[4]

Independent Samples T-Test

Independent Samples T-Test

		Statistic	df	p		Effect Size
COVID Congruent	Student's t	-1.126	38.0	0.267	Cohen's d	-0.356
COVID Incongruent	Student's t	-1.200	38.0	0.238	Cohen's d	-0.379
Pleasant Congruent	Student's t	-0.823	38.0	0.415	Cohen's d	-0.260
Pleasant Incongruent	Student's t	-1.072	38.0	0.291	Cohen's d	-0.339
Unpleasant Congruent	Student's t	-1.641 [a]	38.0	0.109	Cohen's d	-0.519
Unpleasant Incongruent	Student's t	-1.575 [a]	38.0	0.124	Cohen's d	-0.498

[a] Levene's test is significant ($p < .05$), suggesting a violation of the assumption of equal variances

Assumptions

Normality Test (Shapiro-Wilk)

	W	p
COVID Congruent	0.976	0.550
COVID Incongruent	0.962	0.189
Pleasant Congruent	0.958	0.146
Pleasant Incongruent	0.985	0.857
Unpleasant Congruent	0.971	0.386
Unpleasant Incongruent	0.980	0.697

Note. A low p-value suggests a violation of the assumption of normality

Homogeneity of Variances Test (Levene's)

	F	df	df2	p
COVID Congruent	0.418	1	38	0.522
COVID Incongruent	3.390	1	38	0.073
Pleasant Congruent	4.068	1	38	0.051
Pleasant Incongruent	1.415	1	38	0.242
Unpleasant Congruent	8.957	1	38	0.005
Unpleasant Incongruent	6.053	1	38	0.019

Note. A low p-value suggests a violation of the assumption of equal variances

[5]

References

[1] The jamovi project (2022). *jamovi*. (Version 2.3) [Computer Software]. Retrieved from https://www.jamovi.org.

[2] R Core Team (2021). *R: A Language and environment for statistical computing*. (Version 4.1) [Computer software]. Retrieved from https://cran.r-project.org. (R packages retrieved from MRAN snapshot 2022-01-01).

[3] Singmann, H. (2018). *afex: Analysis of Factorial Experiments*. [R package]. Retrieved from https://cran.r-project.org/package=afex.

[4] Lenth, R. (2020). *emmeans: Estimated Marginal Means, aka Least-Squares Means*. [R package]. Retrieved from https://cran.r-project.org/package=emmeans.

[5] Fox, J., & Weisberg, S. (2020). *car: Companion to Applied Regression*. [R package]. Retrieved from https://cran.r-project.org/package=car.
